# Ligand Recognition Determines the Role of Inhibitory B Cell Co-receptors in the Regulation of B Cell Homeostasis and Autoimmunity

**DOI:** 10.3389/fimmu.2018.02276

**Published:** 2018-10-02

**Authors:** Takeshi Tsubata

**Affiliations:** Department of Immunology, Medical Research Institute, Tokyo Medical and Dental University, Tokyo, Japan

**Keywords:** inhibitory B cell co-receptor, CD72, CD22, siglec-G, systemic lupus erythematosus, B-1 cells, Sm/RNP, sialic acid

## Abstract

B cells express various inhibitory co-receptors including CD22, CD72, and Siglec-G. These receptors contain immunoreceptor tyrosine-based inhibition motifs (ITIMs) in the cytoplasmic region. Although many of the inhibitory co-receptors negatively regulate BCR signaling by activating SH2-containing protein tyrosine phosphatase 1 (SHP-1), different inhibitory co-receptors have distinct functional properties. CD22, Siglec-G, and CD72 preferentially regulate tonic signaling in conventional B cells, B-1 cell homeostasis, and development of lupus-like disease, respectively. CD72 recognizes RNA-related lupus self-antigen Sm/RNP as a ligand. This ligand recognition recruits CD72 to BCR in Sm/RNP-reactive B cells thereby suppressing production of anti-Sm/RNP autoantibody involved in the pathogenesis of lupus. In contrast, Siglec-G recognizes α2,3 as well as α2,6 sialic acids whereas CD22 recognizes α2,6 sialic acid alone. Because glycoproteins including BCR are dominantly glycosylated with α2,3 sialic acids in B-1 cells, Siglec-G but not CD22 recruits BCR as a ligand specifically in B-1 cells, and regulates B-1 cell homeostasis by suppressing BCR signaling in B-1 cells. Thus, recognition of distinct ligands determines functional properties of different inhibitory B cell co-receptors.

## Introduction

**Figure 1:**
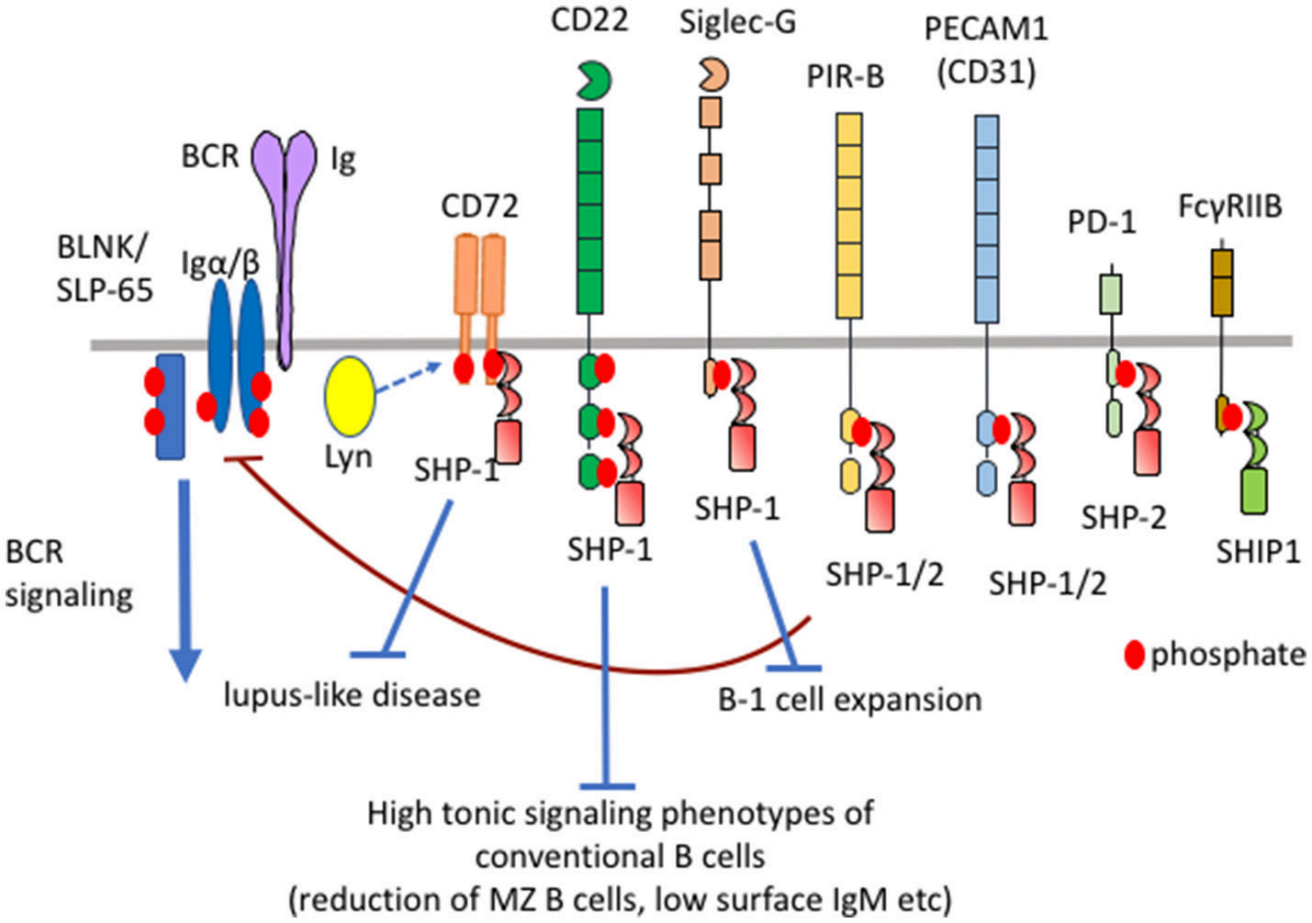
Differential functional properties of inhibitory B cell co-receptors. Mouse B cells express various inhibitory co-receptors such as CD72, CD22, Siglec-G, PIR-B, PEACAM1, PD-1, and FcγRIIB. These receptors contain ITIMs in the cytoplasmic region and recruit SH2-containing phosphatases such as SHP-1, SHP-2, and SHIP-1 upon phosphorylation by Lyn, leading to down-modulation of BCR signaling. Although many of these inhibitory receptors activate SHP-1, CD72, CD22, and Siglec-G inhibits development of lupus-like disease, high tonic signaling phenotypes of conventional B cells, and B-1 cell expansion, respectively.

Antigen-induced signaling through B cell receptor (BCR) plays a central role in B cell responses to antigens (1). BCR also transmits constitutive low level signaling called tonic signaling in the absence of antigen stimulation (2). Tonic signaling regulates B cell survival and development. BCR ligation activates protein tyrosine kinases such as Lyn and Syk, which phosphorylate and activate various down-stream signaling molecules (1). BCR signaling is negatively regulated by various inhibitory co-receptors such as FcγRIIB, Sialic acid-binding Ig-like lectin (Siglec)-10/G (human/mouse ortholog), CD22 (also known as Siglec-2), CD72, PECAM1 (also known as CD31), CEACAM-1, and LILRB/PIR-B (human/mouse ortholog) (3, 4). These inhibitory co-receptors contain immunoreceptor tyrosine-based inhibition motifs (ITIMs) in the cytoplasmic region. ITIMs in FcγRIIB and CD22 are shown to be phosphorylated by Lyn when BCR is ligated. Lyn may also be responsible for phosphorylation of the ITIMs in the other inhibitory co-receptors. Upon phosphorylation, these ITIMs recruit and activate SH2-containing phosphatases such as SH2-containing protein tyrosine phosphatase (SHP)-1, SHP-2, and SH2-containing inositol 5′-phosphatase (SHIP)-1, thereby down-modulating BCR signaling by dephosphorylating signaling molecules activated by BCR ligation (Figure 1). SHIP-1 negatively regulates phosphatidyl inositol 3-kinase (PI-3K)-Akt pathway by dephosphorylating PIP3 generated by PI-3K (5). Studies on B cells deficient in SHP-1 or inhibitory co-receptors demonstrated that proximal signaling molecules of BCR including Lyn, Syk, Igα/Igβ, BLNK/SLP-65 are hyperphosphorylated (6, 7). Because SHP-1 associates with Lyn (8) and Syk (9), these kinases appear to be substrates of SHP-1. The other BCR signaling molecules may be directly or indirectly dephosphorylated by SHP-1. It may be unlikely that SHP-1 activated by different co-receptors dephosphorylate distinct substrates though there is no evidence. CD22 was reported to recruit stimulatory signaling molecules including Syk and phospholipase Cγ (10). However, SHP-1 appears to be the dominant effector of CD22 because CD22 negatively regulates BCR signaling.

FcγRIIB recruits SHIP-1 but not SHP-1 or SHP-2 at phosphorylated ITIMs whereas the other inhibitory B cell co-receptors recruit SHP-1, SHP-2 or both (3). Although the sequence of ITIMs may determine which phosphatase is recruited, the precise mechanism is not yet clear. Although the roles of SHP-2 in B cells is not yet clear, SHP-1 is shown to play crucial roles in the maintenance of B cell homeostasis. B cell-specific conditional SHP-1^−/−^ mice show alterations in the development of conventional B cells, expansion of B-1 cells and development of lupus-like autoimmune disease (11) (Table 1). B cell-specific SHIP-1-deficient mice show similar phenotypes (12). However, FcγRIIB^−/−^ mice show none of these phenotypes (13) although FcγRIIB down-regulates antibody responses and is associated with autoimmune diseases (14). How SHIP-1 is activated to regulate development and homeostasis of B cells is not yet clear. In contrast, deficiency in SHP-1-activating co-receptors CD22, Siglec-G and CD72 causes alterations in the development of conventional B cells (11, 15–18), expansion of B-1 cells (19), and development of lupus-like disease (20, 21), respectively. Thus, SHP-1 activated by different co-receptors regulates distinct B cell phenotypes. Because the roles of the ligands are extensively studied in CD22, Siglec-G, and CD72 among SHP-1-activating B cell co-receptors, I would like to discuss distinct functional properties of different inhibitory co-receptors and the role of ligand recognition in determining their functional properties by focusing on CD22, Siglec-G and CD72.

**Table 1:** B cell phenotypes of mice deficient in inhibitory B cell co-receptors, ligands, and effector phosphatases.

	**Mice**						
**Phenotype**^a^	**SHP-1**^−/−^	**SHIP-1**^−/−^	**CD22**^−/−^	**CD72**^−/−^	**Siglec-G**^−/−^	**Siglec-G R120E**^b^	**Fc**γ**RIIB**
High tonic signaling in conventional B cells^c^	++	++	++	−	−	−	−
B-1 cell expansion	++	++	±	−	++	++	−
Lupus-like disease	++	++	−	++	−	NA^4^	−

a *Both ST6GalI^−/−^ mice deficient in α2,6 sialic acid and CD22 R130E mice expressing CD22 deficient in ligand binding show reduction in BCR signaling in conventional B cells upon BCR ligation*.

b *Deficient in ligand binding*.

c *Reduction in marginal zone B cell population and reduction in the level of cell surface IgM*.

d *Not available*.

## Distinct functional properties of CD22, Siglec-G, and CD72

In Siglec-G^−/−^ mice, the number of B-1 cells in the peritoneal cavity is increased by around 10-folds (19), which is almost equivalent to B-1 cell expansion observed in B cell-specific SHP-1^−/−^ mice (11). In contrast, CD22^−/−^ mice (15, 16), PECAM1^−/−^ mice (22), and PIR-B^−/−^ mice (23) show only modest increase in the number of B-1 cells. Thus, Siglec-G plays a central role in SHP-1-mediated regulation of B-1 cells, whereas other inhibitory co-receptors play an auxiliary or no role in the regulation of B-1 cell homeostasis.

CD22^−/−^ mice as well as B cell-specific SHP1^−/−^ or Lyn^−/−^ mice show various alterations in conventional B cells such as reduction in the number of marginal zone (MZ) B cells and reduction in the level of IgM on the surface of follicular B cells (11, 15–18). Recently, Yasuda et al. demonstrated that IgM^hi^ cells show higher phosphorylation levels of signaling molecules such as Erk and Akt, and better *in vitro* survival compared to IgM^lo^ cells (24), suggesting that the total tonic signaling level required for B cell survival depends on the expression level of BCR. If BCR carries high tonic signaling activity, total tonic signaling level in IgM^lo^ cells may be sufficient for survival. Thus, the reduction in the level of surface IgM in CD22^−/−^ B cells suggests increased tonic signaling activity in the absence of CD22. This notion is also supported by the reduction in MZ B cells in CD22^−/−^ mice because B cells with low tonic signaling are suggested to preferentially differentiate to MZ B cells (25). In contrast, these alterations in conventional B cells are not observed in mice deficient in other inhibitory co-receptors such as CD72.

Almost all CD72^−/−^ mice spontaneously develop lupus-like glomerulonephritis by 6 months of age (21). CD72^−/−^Fas^lpr/lpr^ mice on the C57BL/6 background develop severe lupus-like disease comparable to MRL.Fas^lpr^ mice. Both CD72^−/−^Fas^lpr/lpr^ mice and MRL.Fas^lpr^ mice produce large amounts of autoantibodies such as anti-DNA antibody and develop glomerulonephritis with severe histological changes at 6 months of age. In contrast, mice deficient in other inhibitory co-receptors such as CD22^−/−^ mice and PIR-B^−/−^ mice do not develop autoimmune disease (26, 27). Even by introduction of Fas^lpr^, only a fraction of PIR-B^−/−^Fas^lpr/lpr^ mice develop lupus-like disease at 12 months of age (27). Only a fraction of PECAM1^−/−^ mice and Siglec-G^−/−^ mice develop mild lupus-line disease after 12 months of age (22, 26). Because development of autoimmune disease partly depends on the cleanness of the animal facility, it is not possible to discuss small differences in the disease severity among the different mice housed in different facilities. Nonetheless, CD72^−/−^ mice develop lupus-like disease that is clearly more severe than that developed in mice deficient in other inhibitory co-receptors. Thus, CD72 appears to be a dominant inhibitory B cell co-receptor in the regulation of autoimmune disease.

Taken together, Siglec-G, CD22, and CD72 regulate B-1 cell homeostasis, tonic signaling of conventional B cells, and development of lupus-like disease, respectively (Figure 1; Table 1), suggesting that different inhibitory B cell co-receptors regulate distinct B cell phenotypes.

## Role of ligands in determining functional properties of inhibitory B cell co-receptors

Most of the inhibitory co-receptors recognize endogenous ligands (Table 2). Role of the endogenous ligands in determining the functional properties of inhibitory co-receptors was first demonstrated in FcγRIIB already in 1990s. FcγRIIB inhibits BCR signaling when co-ligated with BCR. Binding of immune complexes composed of antigens and IgG with BCR induces co-ligation of FcγRIIB and BCR, thereby down-regulating BCR signaling and antibody responses to the antigens (28, 29). In contrast, roles of endogenous ligands of SHP-1-activating inhibitory B cell co-receptors were not clear until a few years ago.

**Table 2:** Inhibitory co-receptors and their ligands.

**Inhibitory co-receptors**	**Expression**	**Ligands**	**Role of ligands^a^**	**Expression of ligands**
CD22	Constitutive	α2,6 sialic acid	Inhibitory	Ubiquitous
CD72	Constitutive	Sm/RNP	Stimulatory	Released from dead cells
		CD100 (Sema4D)	Inhibitory	Various hematopoietic and non-hematopoietic cells
Siglec-G	Constitutive	α2,3 sialic acid	Stimulatory	Ubiquitous, B1 cells >> conventional B cells
		α2,6 sialic acid	Stimulatory	Ubiquitous
PIA-B	Constitutive	MHCI	Stimulatory	Ubiquitous
PECAM1	Constitutive	PECAM1	?	Endothelial cells, hematopoietic cells
		α2,6 sialic acid	?	Ubiquitous
PD-1	Inducible	PD-L1	Stimulatory	Hematopoietic cells, various non-hematopoietic cells
		PD-L2	Stimulatory	Macrophages, DCs, mast cells, B-1 cells
FcγRIIB	Constitutive	IgG	Stimulatory	

a *Inhibitory or stimulatory role in co-receptor-mediated signal inhibition*.

CD72 is a type II membrane molecule containing a C-type lectin-like domain (CTLD) in the extracellular region. The ligand of CD72 was initially reported to be CD5, although this result has not been reproduced (30). Later, CD100 (also known as Semaphorin-4D) was shown to be an inhibitory ligand of CD72 (31). The functional significance of this inhibitory ligand is not yet clear. We demonstrated that the extracellular CTLD of CD72 specifically recognizes the lupus self-antigen Sm/RNP as a ligand (32). Sm/RNP is a major RNA-containing lupus self-antigen, and a ligand of the endosomal RNA sensor TLR7 (33). Because TLR7 but not the DNA sensor TLR9 is essential for development of lupus-like disease in multiple mouse models (34), autoimmune response to RNA-related self-antigens such as Sm/RNP appears to be crucial in development of SLE.

**Figure 2:**
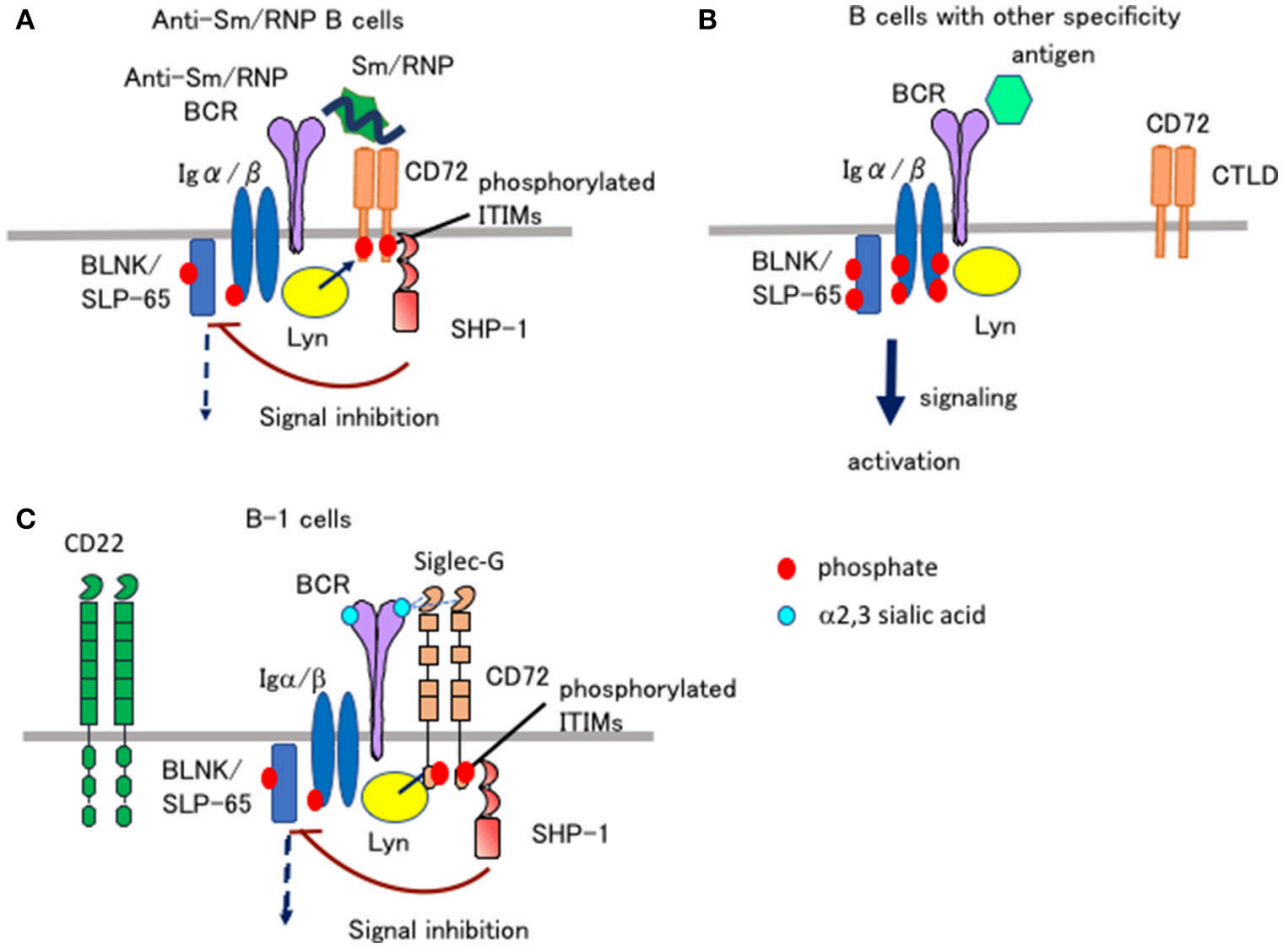
Ligand recognition determines the functional properties of CD72 and Siglec-G. **(A,B)** CD72 specifically inhibits BCR signaling in Sm/RNP-reactive B cells by recognizing Sm/RNP as a ligand. When Sm/RNP interacts with Sm/RNP-reactive BCR, CD72 is recruited to BCR by recognition of Sm/RNP **(A)**. CD72 ITIM is then phosphorylated by Lyn and recruits SHP-1. This leads to suppression of BCR signaling in Sm/RNP-reactive B cells thereby inhibiting production of anti-Sm/RNP antibody crucial for development of lupus. When other antigens that do not bind to CD72 interact with BCR, CD72 is kept away from BCR and does not inhibit BCR signaling **(B)**. **(C)** Siglec-G inhibits BCR signaling in B-1 cells by recognizing α2,3 sialic acid as a ligand. Because glycoproteins in B-1 cells are dominantly glycosylated with α2,3 sialic acid, Siglec-G constitutively associates with BCR by recognizing α2,3 sialic acid expressed on BCR in B-1 cells, thereby down-modulating BCR signaling (35). CD22 does not regulate BCR signaling in B-1 cells because CD22 recognizes α2,6 sialic acid but not α2,3 sialic acid.

When BCR is ligated by Sm/RNP, CD72^−/−^ B cells show augmented Ca^2+^ and proliferative responses compared to CD72^+/+^ B cells (32). In contrast, Ca^2+^ and proliferative responses to a control antigen in CD72^−/−^ B cells are comparable to that in CD72^+/+^ B cells. This result suggests that CD72 specifically down-regulates BCR signaling when BCR is ligated by Sm/RNP. When Sm/RNP binds to BCR expressed on the surface of Sm/RNP-reactive B cells, CD72 appears to be recruited to BCR because of its binding to Sm/RNP (Figure 2A). Antigen-mediated recruitment of CD72 to Sm/RNP-reactive BCR may induce phosphorylation of the CD72 ITIM by BCR-associated Lyn, leading to SHP-1-mediated suppression of BCR signaling. In contrast, CD72 may not be recruited to BCR when BCR interacts with the other antigens that do not bind to CD72 (Figure 2B). Thus, CD72 negatively regulates BCR signaling induced by Sm/RNP but not the other antigens, thereby specifically inhibits activation of B cells reactive to Sm/RNP. In CD72^−/−^ mice, Sm/RNP activates B cells reactive to Sm/RNP probably by inducing both BCR signaling and TLR7 signaling, leading to the production of anti-Sm/RNP antibody crucial for development of lupus. CD72 appears to inhibit development of lupus by inhibiting activation of Sm/RNP-reactive B cells.

Both CD22 and Siglec-G are members of the Siglec family, and recognize sialic acids as a ligand (36). CD22 specifically recognizes α2,6 sialic acid, whereas Siglec-G broadly recognizes both α2,3 and α2,6 sialic acids. Previously, Nitschke and his collaborators addressed how Siglec-G but not CD22 strongly regulates BCR signaling in B-1 cells and B-1 cell homeostasis (35), although both Siglec-G and CD22 are expressed by both B-1 cells. They demonstrated that the Siglec-G mutant deficient in ligand binding no longer associates with BCR nor down-regulates BCR signaling, suggesting that Siglec-G associates with BCR by recognizing sialic acid located in BCR thereby inhibiting BCR signaling. They further demonstrated that B-1 cells express α2,3 sialic acid at much higher level than conventional B cells. Recognition of α2,3 sialic acid by Siglec-G induces association of Siglec-G and BCR specifically in B-1 cells, which may induce phosphorylation of the Siglec-G ITIM by Lyn and activation of SHP-1 required for inhibition of BCR signaling (Figure 2C).

Although CD22 regulates tonic signaling, how ligand recognition of CD22 is involved in this function is not yet clear. As is the case for Siglec-G in B-1 cells, CD22 is shown to be associated with BCR by recognizing a sialylated ligand in conventional B cells (37). However, studies with mice deficient in ST6GalI, the sialyl transferase required for the synthesis of α2,6 sialic acid, and those with mice expressing a mutant CD22 that do not recognize α2,6 sialic acid showed that endogenous ligands rather down-modulate suppressive activity of CD22 (38–40). These findings are contradictory to the model in which ligand recognition induces CD22-mediated signal inhibition. Whether ligand recognition is involved in the functional properties of CD22 needs to be further studied. Other inhibitory co-receptors also recognize endogenous ligands (3). PIR-B is known to interact with MHC I (41). Because PIR-B phosphorylation is modestly reduced in β2m^−/−^ splenocytes (42), interaction of PIR-B with MHC-I may facilitate PIR-B-mediated signal inhibition. PECAM1 and CEACAM-1 (4) show homotypic interaction with trans-ligands, and PECAM1 was also shown to recognize sialic acids (43). How ligand recognition regulates the functional activities of these inhibitory co-receptors is not yet clear (Table 2).

## Conclusions and future perspective

The inhibitory B cell co-receptors CD22, CD72, and Siglec-G regulate distinct B cell functions: CD22 regulates tonic signaling in conventional B cells, Siglec-G regulates B-1 cell homeostasis and CD72 regulates autoimmunity. Recognition of Sm/RNP induces association of CD72 with BCR in B cells reactive to Sm/RNP whereas recognition of α2,3 sialic acid induces association of Siglec-G with BCR in B-1 cells. Thus, different inhibitory co-receptors associate with BCR in distinct B cell populations depending on the ligand recognition of inhibitory co-receptors, thereby regulating distinct B cell functions, i.e., development of lupus-like disease by CD72 and B-1 cell homeostasis by Siglec-G. Recognition of endogenous ligands thus determines the B cell phenotypes regulated by CD72 and Siglec-G. Further determination of ligands of inhibitory co-receptors and elucidation of the roles of ligand recognition may advance our understandings on how inhibitory co-receptors regulate development and differentiation of B cells and suppress activation of pathological B cells. These studies may provide clues in understanding pathogenesis of immunological diseases.

## Author contributions

TT conceived of this mini review and wrote the manuscript.

### Conflict of interest statement

The author declares that the research was conducted in the absence of any commercial or financial relationships that could be construed as a potential conflict of interest.
